# *JAK2V617F* positive polycythemia vera with paroxysmal nocturnal hemoglobinuria and visceral thromboses: a case report and review of the literature

**DOI:** 10.1186/s12959-021-00269-8

**Published:** 2021-03-10

**Authors:** Sevastianos Chatzidavid, Nefeli Giannakopoulou, Panagiotis Theodorou Diamantopoulos, Eleni Gavriilaki, Panagiota Katsiampoura, Eleftheria Lakiotaki, Stratigoula Sakellariou, Nora-Athina Viniou, Georgios Dryllis

**Affiliations:** 1 Hematology Unit, First Department of Internal Medicine, Laikon General Hospital, National and Kapodistrian University of Athens, Athens, Greece; 2 Hematology Department – Bone Marrow Transplant Unit, George Papanicolaou Hospital, Thessaloniki, Greece; 3 First Department of Pathology, Laikon General Hospital, National and Kapodistrian University of Athens, Athens, Greece

**Keywords:** Visceral thrombosis, Paroxysmal nocturnal hemoglobinuria, Polycythemia vera, Myeloproliferative neoplasm

## Abstract

**Background:**

Polycythemia vera (PV) is characterized by red cell mass expansion in the peripheral blood and can be complicated with thrombosis, bleeding, evolution to acute myeloid leukemia (AML) or a fibrotic phase. Paroxysmal nocturnal hemoglobinuria (PNH) in an acquired clonal haematopoietic stem cell disorder associated with chronic intravascular hemolysis, venous thrombosis, defective hematopoiesis, frequent episodes of infection and, rarely, leukemic transformation. Herein, we report an interesting case of a patient with coexistence of PNH clones and a *JAK2V617F* positive PV, with unusual thromboses without hemolysis.

**Case presentation:**

A 51-year-old woman presented with increased levels of hematocrit, multiple liver, spleen, and left kidney infarctions and ascites; further investigation revealed a *JAK2V617F*-positive polycythemia vera and the presence of a significant PNH population (more than 90% CD55– CD59– cells among both granulocytes and red blood cells). Interestingly, the patient has experienced severe thrombotic events without any signs or symptoms of hemolysis.

**Conclusions:**

This case raises questions over uncharted aspects of the PNH etiopathogenesis and its potential association with myeloproliferative neoplasms (MPN) and highlights the difficulty of diagnosing and managing patients with more than one potentially thrombophilic conditions, especially with established and severe thromboses.

## Background

Myeloproliferative neoplasms (MPN) usually exhibit terminal myeloid cell expansion in the peripheral blood [1]. Over time, the clinical course of MPNs can be complicated with thrombosis, bleeding, and evolution to acute myeloid leukemia (AML) or a fibrotic phase of the disease [2, 3]. Polycythemia vera (PV) is distinguished clinically from other MPNs by the elevation of the red blood cell mass over 125% of normal expressed or as an increase in hematocrit (Hct) of more than 49% for men and 48% for women [2]. Most patients with PV are diagnosed incidentally when elevated hemoglobin (Hgb) or Hct is noted on a complete blood count. Others present with disease-related symptoms (e.g., headache, dizziness, visual disturbances, pruritus, early satiety) or complications (e.g., thrombosis, bleeding) [4].

Paroxysmal nocturnal hemoglobinuria (PNH) in an acquired clonal hematopoietic stem cell disorder characterized by chronic intravascular haemolysis, venous thrombosis, defective hematopoiesis, frequent episodes of infection and, rarely, leukemic transformation. The mechanism of hemolysis appears to be an unregulated complement activation on the abnormal red cell surface, due to reduction or absence of regulatory membrane molecules protecting cells from the membrane attack complex of complement mediated lysis, such as CD55 and CD59 [5–7]. While PNH is a non-neoplastic clonal disorder, it has long been closely linked to clinical entities such as aplastic anemia (AA) and myelodysplastic syndromes (MDS) [5]. Occasionally, CD55 and/or CD59 deficient clones are detected in non-cytopenic patients and patients with no apparent bone marrow failure syndromes and in about 4,1% in healthy volunteers (1.6, 0.8% and 1,6% with CD55−/CD59-, CD55−/CD59+, and CD55+/CD59- cells respectively) [8].

In this paper we report an interesting case of a female patient with coexistence of PNH clones and a *JAK2V617F* positive PV, with unusual thromboses and not overt hemolysis.

## Case presentation

A 51-year-old woman with a medical history of cholecystectomy and beta thalassemia trait presented to the emergency department because of severe epigastric pain during the last 3 days. The patient reported findings of high hematocrit levels in the previous months (Hct ≈ 48%) without any further evaluation and episodes of pruritus in her extremities and trunk during the last 7 months. The initial blood tests revealed an elevated Hct level of 44.8% with Hgb levels of 14.4 g/dl, a white blood cell (WBC) count of 4.81 × 10^9^/L – with 55% neutrophils - and a platelet (PLT) count of 227 × 10^9^/L. Aspartate aminotransferase (AST), alanine aminotransferase (ALT) and lactate dehydrogenase (LDH) were markedly elevated, while alkaline phosphatase (ALP) and gamma-glutamyltransferase (γGT) were significantly elevated. The other biochemical markers were within normal limits (Table 1).

**Table 1:** Laboratory findings timeline

Laboratory parameter (unit)	At presentation	3 weeks under HU and LMWH	10 months after eculizumab initiation	Normal ranges
Hgb (g/dL)	1.4	17.6	10.9	14.0–16.5
Hct (%)	44.8	55.2	36.1	38.0–52.0
MCV (fL)	83.5	81.2	74.3	80–96
MCH (pg)	26.4	25.9	22.4	27–31
WBC (×10^9^/L)	4.8 (55% N)	13.0 (83% N)	7.0	4.5–11.0
PLT (×10^9^/L)	227	177	241	140–440
INR	1.73	5.50	2.31	0.90–1.20
APTT (sec)	40.8	62.2	NA	29–40
Fibrinogen (mg/dL)	456	305	NA	180–400
D-Dimers (μg/mL)	3.83	3.35	NA	< 0.5
Urea (mg/dL)	29	23	29	15–43
Creatinine (mg/dL)	0.55	0.74	0.59	0.7–1.2
Total bilirubin (mg/dL)	0.52	1.41	0.56	0.3–1.2
Direct bilirubin (mg/dL)	0.38	0.90	0.28	0–0.3
LDH (U/L)	399	280	327	135–225
ALP (U/L)	940	1064	468	40–129
γ-GT (U/L)	297	471	125	8–61
AST (U/L)	82	222	22	15–40
ALT (U/L)	175	290	20	< 41
CRP (mg/dL)	32.1	35.60	4.41	0–5
Ferritin (ng/mL)	160.7	95.1	20	15–150
Fe (μg/dL)	34	50	8.6	37–145
Serum Erythropoietin (μIU/mL)	11.99	NA	NA	3.22–31.9

Normal ranges are listed in the last column

*Hgb* Hemoglobin, *Hct* Hematocrit, *MCV* Mean corpuscular volume, *MCH* Mean corpuscular haemoglobin, *WBC* White blood cells, *PLT* Platelets, *INR* International normalized ratio, *APTT* Activated partial thromboplastin time, *LDH* Lactate dehydrogenase, *ALP* Alkaline phosphatase, *γ-GT* Gamma-glutamyltransferase, *AST* Aspartate aminotransferase, *ALT* Alanine aminotransferase, *CRP* C-reactive protein, *HU* Hydroxyurea, *LMWH* Low molecular weight heparin

**Fig. 1:**
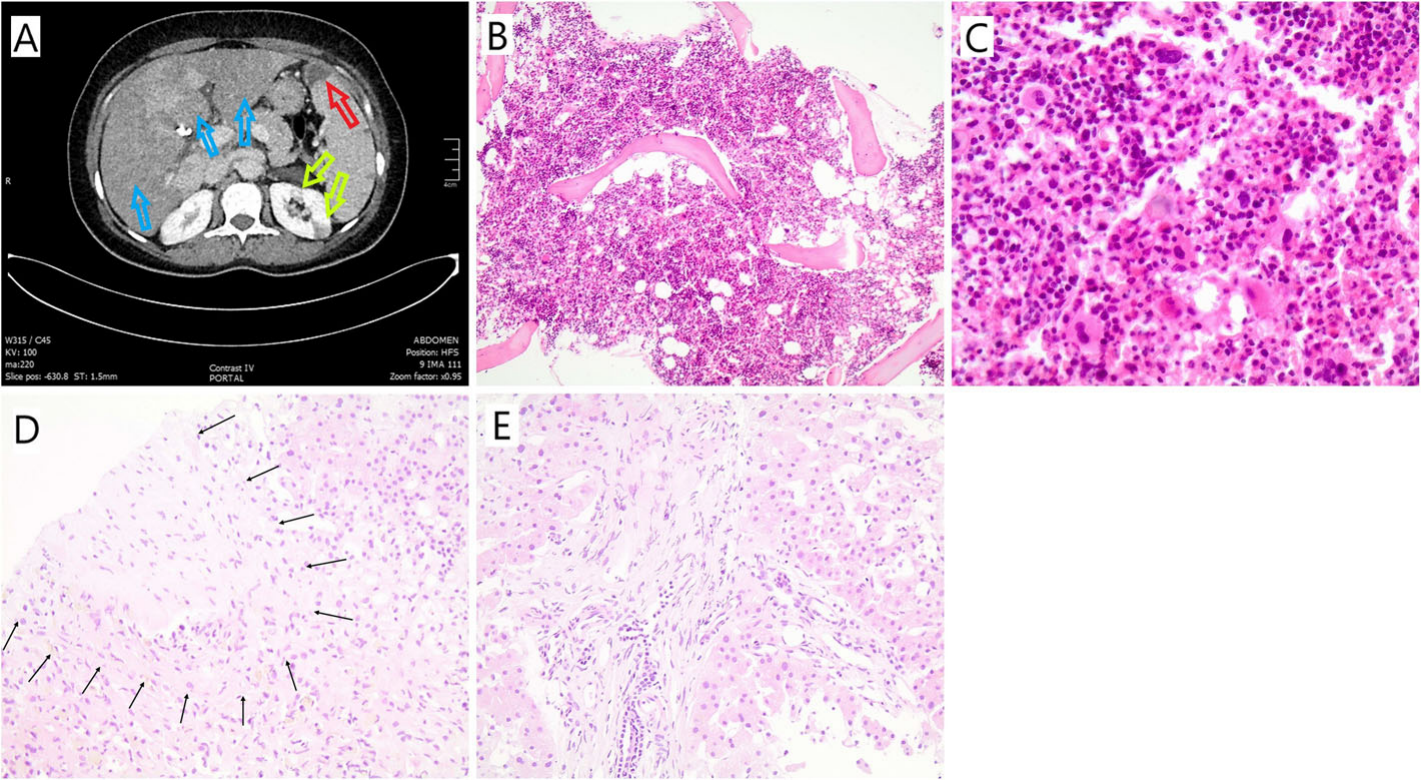
**a** Abdominal Computed Tomography of the patient’s multiple liver (yellow arrows); spleen (red arrow) and left kidney (green arrows) infarctions. **b** Bone marrow biopsy (hematoxylin-eosin stain, X100): the bone marrow is markedly hypercellular for the age of the patient. **c** Bone marrow biopsy (hematoxylin-eosin stain, X400): trilineage hyperplasia with increased numbers of erythroid precursors and prominent megakaryocytes of different size. **d** Liver biopsy (hematoxylin-eosin stain, X200): Terminal hepatic venule with fibrous occlusion of the lumen and fibrous thickening of the subendothelial connective tissue (black arrows delineating the venule periphery). **e** Liver biopsy (hematoxylin-eosin stain, X200): Mild lymphocytic infiltrates with periportal fibrosis and sinusoidal dilatation of the adjacent parenchyma

Initial investigation with an abdominal ultrasound showed liver and spleen enlargement (17.5 cm and 15 cm, respectively), with grade 1 ascites, whereas a Computed Tomography (CT) scan revealed multiple liver, spleen, and left kidney infarctions (Fig. 1a). Splenic or portal vein thromboses were not found. A transthoracic echocardiogram revealed no additional findings. Furthermore, an upper endoscopy identified the presence of portal hypertensive gastropathy. The serum ascites albumin gradient (SAAG) was 1.7 g/dl, compatible with portal hypertension.

An autoimmune inflammatory disorder panel showed that antinuclear antibodies (ANA) and anti - extractable nuclear antigen (ENA) - La antibodies were positive. Thrombophilia workup revealed homozygosity for the C677T *MTHFR* (methylene-tetrahydrofolate-reductase) gene mutation with normal blood homocysteine levels (Table 2).

**Table 2:** Autoimmune inflammatory disorder panel and thrombophilia workup

Autoimmune inflammatory disorder panel		Thrombophilia workup	
ΑΝΑ	> 1:640 AC-4	factor V (Leiden) gene mutation	negative
anti-ENA-La	positive	G20210A factor II gene mutation	negative
anti-ENA-Ro	negative	AT deficiency	negative
anti-ENA-RNP	negative	protein C deficiency	negative
anti-ENA-Sm	negative	protein S deficiency	negative
p-ANCA	negative	*MTHFR*	C677T homozygosity
c-ANCA	negative	blood homocysteine level	7 μmol/L (NR 5–15 μmol/L)
anti-dsDNA	negative	aCL and anti-β 2-GPI antibodies	negative
AMA	negative		
ASMA	negative		

*ANA* Antinuclear antibody, *AC-4* Anti cell-4 pattern, *ENA* Extractable nuclear antigen, *RNP* Ribonucleoprotein, *Sm* Smith, *p-ANCA* Perinuclear anti-neutrophil cytoplasmic antibodies, *c-ANCA* Antineutrophil cytoplasmic antibodies, *dsDNA* Double stranded DNA, *AMA* Antimitochondrial antibodies, *ASMA* Anti-smooth muscle antibody, *AT* Antithrombin, *MTHFR* Methylenetetrahydrofolate reductase, *aCL* Anti-cardiolipin, *β2-GPI* Beta-2-glycoprotein I, *NR* Normal range

A whole blood allele-specific polymerase chain reaction (PCR) analysis showed that the patient carried the *JAK2V617F* mutation. The serum erythropoietin (EPO) level was within normal range (Table 1). A bone marrow biopsy revealed a hypercellular bone marrow (90%), with erythroid hyperplasia, mild dyserythropoiesis, mild granulocytic hyperplasia with no dysplastic features and no increase in the blast count (Fig. 1b-c). Considering all the above the patient was diagnosed as having masked PV complicated with multiple visceral thromboses.

The patient was initially treated with hydroxyurea at 500 mg qd po and enoxaparin at a dose of 1 mg/kg subcutaneously (SC) q12h which corresponds to 6000 IU q12h according to the patient’s weight. A mild decrease of the Hct, AST, ALT, γ-GT, and ALP was initially observed before a second rise during an episode of abdominal pain and ascites causing marked abdominal distension 3 weeks after therapy introduction (Table 1). Radiological reassessment with an abdominal CT angiography detected thrombosis of the hepatic veins and a liver biopsy was performed which was consistent with the diagnosis of Budd – Chiari syndrome (Fig. 1d-e). A peripheral blood flow cytometry analysis was performed, revealing a 91.8% of granulocytes, 45.5% of monocytes and 90.6% of erythrocytes CD55 and CD59-deficient population, suggestive of PNH. The patient’s history did not reveal any indications of hemolytic anemia and associated symptoms, such as fatigue, jaundice, or urine discoloration. At the time of diagnosis, the reticulocyte count, haptoglobin, and indirect bilirubin level were within normal range. A direct antiglobulin (Coombs) test was negative and urine samples were negative for hemoglobin or hemosiderin.

Because of suboptimal response to enoxaparin and hydroxyurea, enoxaparin was switched to acenocumarol, while, due to the appearance of large PNH clones, initiation of complement inhibition treatment with eculizumab was decided. Before eculizumab initiation, the patient was vaccinated with a serogroup B meningococcal vaccine.

After 2 months on hydroxyurea, acenocoumarol, and eculizumab the Hct and Hgb levels decreased, cholestatic enzyme levels were markedly improved, and the liver enzymes were within normal range. Ascites and abdominal pain completely resolved. The laboratory findings after 10 months of treatment are listed in Table 1.

## Discussion and conclusion

Patients with PV have an increased risk of thrombotic complications such as cerebrovascular event, myocardial infarction, superficial thrombophlebitis, deep vein thrombosis (DVT), pulmonary embolism or hemorrhage, as well as microcirculatory disorders, such as erythromelalgia, visual and neurologic symptoms. In a large international study, an arterial or venous thrombotic complication or major hemorrhage was noted prior to or at the time of diagnosis in 16, 7, and 4% of patients with PV [4]. Hyperviscosity may also contribute to the pathogenesis of thrombosis in PV with *JAK2V617F* mutation [9].

A high percentage of patients with idiopathic hepatic (eg, Budd-Chiari syndrome) or portal vein thrombosis, but not those with idiopathic lower extremity DVT [9–12], bear the *JAK2V617F* mutation suggestive of an occult MPN [13–16]. Approximately 40% of patients with visceral vein thrombosis is *JAK2V617F* mutated but the same studies show that another risk factor for thrombosis is present, either a hypercoagulable disorder or a predisposing condition [17]. Although the mechanisms involved in this hypercoagulable state are unclear, abnormalities in blood viscosity, platelets, and leukocytes have been implicated [18]. Major thrombotic events can occur in patients who otherwise have few clinical and laboratory features of PV. Examples include the Budd - Chiari syndrome and portal, splenic, or mesenteric vein thrombosis [19], but portal hypertension and hypersplenism may mask the increase in blood cell counts [13, 17, 20–23]. Why thrombosis occurs in atypical locations rather than typical ones is not well understood. Relatively low flow rates in intra-abdominal vessels have been postulated as a potential mechanism. Thus, PV should be excluded in patients with these diagnoses, particularly in women under the age of 45.

PNH patients usually present with chronic intravascular hemolysis and hemoglobinuria accompanied by leukopenia and thrombocytopenia. However, PNH may sometimes present with diverse and tricking clinical features [18]. Acute and chronic venous thrombosis is the leading cause of death in patients with PNH. The pathogenesis of thrombosis in PNH is multifactorial and incompletely understood. However, there are several theories under investigation [24, 25]. Complement inhibition seems to reduce both hemolysis and thrombotic complications [26]. In addition, the risk of thrombosis correlates with the size of the PNH clone; however, it is not well understood whether this correlation reflects the degree of hemolysis or another mechanism [24]. It seems that the risk of a thrombotic event is directly related to the size of the PNH clone, and in particular to the percentage of granulocytes with a lack of glycosyl phosphatidylinositol (GPI)-anchored proteins. A size over 50% is associated with a thrombosis rate of about 45%, while a size below 50% with a thrombosis rate of about 5.8%, still higher than that of the general population (5 thrombotic events occur per 10,000 patients per year) [27]. The risk increases by 1.64-fold for each additional increase in size by 10%, so that patients with more than 70% deficiency have a 12 times higher risk than those with a 20% deficiency [28]. The PNH clone of the platelets is significantly correlated with that of the granulocytes and appears to contribute to the thrombotic risk [29]. PNH cases indicate that thrombotic episodes, even in patients with large clones, may occur with or without minimal hemolysis [30].

However, the coexistence of PNH and PV has never been actively investigated. By reviewing the literature we identified 37 cases where PNH may be associated with an MPN [5, 31, 32], and two cases with thrombosis but no hemolysis, similarly to the case we present here [3].

PNH clones are present in several hematological diseases. In aplastic anemia (AA) deficiency in both CD55 and CD59 molecules has been detected in 33.3% of patients, while in MDS patients the corresponding percentage was 16.5% (50% with a hypoplastic bone marrow) and in healthy individuals 4.1% [5, 7]. The association between PNH and MPNs is rare and difficult to identify. The coexistence of PNH and primary myelofibrosis (PMF) with a JAK2 mutation has been reported by several authors [5, 33–36], while PNH coexistence with an MPN, possibly chronic neutrophilic leukemia has also been described [37]. Although PNH defects have been described in five series of patients with any type of MPN [50% of patients with PMF [31] and 59% of 22 patients with an MPN not further specified] [38–42] only one patient with CML and one with PMF among 50 patients had a positive sucrose lysis test of 5% or greater with no clinical evidences of thrombosis or hemolysis [43].

Two cases in which MPN can be associated to a PNH clone without overt hemolysis at diagnosis have been reported. These PNH clones were detected in *JAK2V617F*-mutated patients and were characterised by GPI-anchored proteins deficiency ranging between 0.05 and 99% [3, 39]. These reports clearly showed that the *JAK2V617F* mutation was not in the germline, but it coexisted within the PNH clone [3]. This association may be attributed to the PNH clone arising either in the *JAK2V617F*-mutated population or in parallel to the *JAK2V617F*-mutated population.

The above referenced studies demonstrate that CD59 mediated signals via antibody cross-linking may induce the activation of protein-tyrosine kinases leading to a rapid increase in the tyrosine phosphorylation of several proteins like p120 [44]. The identification of such CD59-mediated signals may explain why patients with PNH might be susceptible to proliferative disorders and may provide a possible link between PNH and MPN. Moreover, other studies supported that *PIG-A* mutation can occur alone or be followed by one or more secondary subclonal mutations [45]. This is possibly how a PNH clone can expand in parallel with an MPN clone.

Both MPN and PNH are thrombophilic conditions, with a high risk of developing major thromboses in approximately 50% of the patients [30, 46]. The thrombotic predisposition in PNH can be attributed to different factors such as nitrogen oxide depletion, complement activation, and to a large number of inflammatory cytokines, without the presence of overt hemolysis [3, 30].

In our patient, initial diagnosis was that of a masked PV due to low Hct and normal EPO levels at presentation. It is known that some patients with PV who were diagnosed with Budd-Chiari syndrome presented with elevated serum EPO levels possibly explained by hypoxic liver injury and hepatocyte necrosis [47]. In our case, the failure of the treatment and the new findings from further investigation forced us to reevaluate the situation and consider the diagnosis of PNH. After the detection of the PNH clones we had to consider their potential contribution to the clinical presentation of the patient and to the treatment strategy. Although PNH clones of any size have been described in several patients with MPN, their size, potential prothrombotic role, and the failure of cytoreductive treatment were factors taken into account for the decision of eculizumab administration. Regarding Hct and Hgb decrease after eculizumab initiation, it is worth noting that the ferritin levels also decreased in parallel to the mean corpuscular volume (MCV) and the mean corpuscular hemoglobin (MCH) suggesting an iron deficiency contribution to Hct and Hgb lowering (Table 1).

As discussed in the literature, PNH clones may be a confusing and even deceiving finding among patients with any type of MPN especially in the absence of hemolytic anemia. The infrequency of the disease and the nonspecific clinical findings can result in critical delays in the diagnosis and treatment highlighting the importance of prompt and accurate diagnosis. However, testing for PNH in every patient with an MPN could not be applied as a general direction. Patients with an MPN who will likely benefit from a PNH screening should include:

- Those presenting with signs of hemolytic anemia with or without thrombosis
- Those with unexplained cytopenia
- Those presenting with a thrombosis at unusual sites (such as visceral veins or cerebral sinuses) [48, 49] or recurrent episodes of thrombosis under cytoreductive and prophylactic anticoagulation in the absence of any additional risk factors.

Young patients in any of the above categories should be managed with extra caution as the median age of PNH diagnosis is 35 to 40 years. In cases where both diseases coexist or there is a strong suspicion of coexistence, further investigation should follow. The real challenge for the clinician is in determining the degree of involvement of each disease in the clinical presentation and the arising complications. The response to prior treatment and the size of the clones could determine the therapeutic decisions.

Patients with MPN and venous thrombotic events should be initiated at appropriate doses of anticoagulation with LMWH, fondaparinux, unfractionated heparin (UFH), oral factor Xa inhibitors or direct thrombin inhibitors along with cytoreductive agents such as hydroxyurea. Long-term anticoagulation therapy is given after the initial few days for a period which is individualized per patient. Agents for long-term use include oral anticoagulants (e.g. factor Xa inhibitors, direct thrombin inhibitors, Vitamin K antagonists (VKA), and subcutaneous anticoagulants such as LMWH and fondaparinux. Choosing among these options depends upon clinician experience, bleeding risk stratification, patient comorbidities and preferences, and the cost of treatment.

The most effective treatment strategy in patients with PNH and acute thrombotic events consists of initiation of anticoagulation (in the absence of major contraindications) starting with heparin and anticomplement therapy (ACT) with eculizumab or ravulizumab (where available). Long term anticoagulation with VKAs is generally recommended as there are not enough data on the experience with the newer oral anticoagulants in PNH. Recurrent thromboses and worsening of existing thromboses are frequent complications in PNH. In patients who do not have access to ACT, anticoagulation should be started immediately, and they should be referred for potential hematopoietic cell transplantation. ACT is also recommended in patients who develop disabling fatigue, transfusion dependence, frequent symptoms of smooth muscle dystonia, significant renal insufficiency, or other end-organ manifestations of PNH. Additional inhibition therapy may be initiated within 24 h of the occurrence of any thrombotic event, to reduce the risk of extension of the thrombotic site or its relapse [30].

The therapeutic interventions in subjects with MPN who also have PNH should include addition of ACT to the treatment strategy. According to all previous data, a suggested therapeutic algorithm for patients with thrombosis and documented co-existence of PNH and MPN is the following.

If ACT (eculizumab or ravulizumab) is not available, initiation of cytoreductive treatment (ie hydroxyurea) and anticoagulation starting with heparin and continuing in the long term with VKAs could be suggested. Major contraindications and comorbidities should be taken into account and the patient should be closely monitored and informed of the risk of bleeding and thrombosis relapse [6, 7, 50]. If ACT is available, co-administration of cytoreductive treatment, ACT, and heparin continuing in the long term with VKAs is a possible option [38].

The optimal duration of anticoagulant therapy in this setting is unknown because of the rarity of the coexistence. Data from registries on PNH alone suggest that it may be safe to stop anticoagulation in PNH patients on eculizumab [51]. Therefore, more experience and long follow-up is necessary before considering discontinuing anticoagulation in patients with MPN and PNH. Until then, close monitoring, weighing the advantages and disadvantages of long-term anticoagulation, management of other comorbidities, and discussion with the patient should be followed [51].

Allogeneic bone marrow transplantation should be considered in patients who do not have access to ACT when other measures have failed [30]. Corticosteroids alone or immunosuppressive therapy such as danazole, antithymocyte globulin or cyclosporine have been used as alternative agents in PNH although the underlying mechanisms remain unclear [44] and their effectiveness is still largely unknown. Iron supplementation when loss through the urine is significant and folic acid to compensate for hemolysis should be additionally given.

In conclusion, the clinical significance of the coexistence of PNH and MPN is interesting due to its rarity while at the same time it has not been investigated in detail. Given the heterogeneity of clinical presentation and the complex pathophysiology of both diseases, further studies need to be conducted to assess the true nature of this association. Treatment options must be confirmed in prospective trials. Physicians treating patients with MPNs should always keep in mind that rare diseases such as PNH can coexist and adjust the management on a case by case plan.

## Data Availability

The datasets used and/or analyzed during the current study are available from the corresponding author on reasonable request. Citations are included in the reference list.
